# Zinc(II) and Nickel(II) Benzoate Complexes from the Use of 1-methyl-4,5-diphenylimidazole

**DOI:** 10.1155/2010/178034

**Published:** 2010-07-21

**Authors:** Konstantina A. Kounavi, Manolis J. Manos, Anastasios J. Tasiopoulos, Spyros P. Perlepes, Vassilios Nastopoulos

**Affiliations:** ^1^Department of Chemistry, University of Patras, 265 04 Patras, Greece; ^2^Department of Chemistry, University of Cyprus, 1678 Nicosia, Cyprus

## Abstract

Two new complexes, [Zn(O_2_CPh)_2_(L)_2_]*·*2MeOH (**1** *·*2MeOH) and [Ni_2_(O_2_CPh))_4_(L)_2_]*·*2MeCN (**2** *·*2MeCN), have been synthesized and characterized by X-ray analysis in the course of an ongoing investigation of the M^II^/X^−^/L [M^II^ = Co, Ni, Cu, Zn; X^−^ = Cl^−^, Br^−^, I^−^, NCS^−^, NO_3_^−^, N_3_^−^, PhCO_2_^−^; L = 1-methyl-4,5-diphenylimidazole] reaction system, aiming at understanding and assessing the relative strength and the way in which the intermolecular interactions control the supramolecular organization of these compounds. In the mononuclear complex **1** *·*2MeOH, the benzoate ion acts as a monodentate ligand resulting in a distorted tetrahedral N_2_O_2_ coordination environment. Complex **2** *·*2MeCN exhibits a dinuclear paddle-wheel structure; each Ni^II^ has a square pyramidal NiNO_4_ chromophore with four benzoate oxygens in the basal plane and the pyridine-type nitrogen atom of one ligand L at the apex. The structure of **1** *·*2MeOH is stabilized by intramolecular *π*-*π* interactions between aromatic rings of adjacent 4,5-diphenylimidazole moieties; it is a feature also evidenced in similar compounds of the type [MX_2_L_2_].

## 1. Introduction

Imidazole and its derivatives have played a formative role in the development of coordination chemistry [1, 2]. Many hundreds of neutral complexes and complex ions containing imidazoles have been prepared and characterized. The variety of spectroscopic properties and stoichiometries observed led to an improved understanding of the geometry and bonding in complexes and provided a touchstone for bonding theories. Imidazoles are particularly interesting ligands in bioinorganic [3, 4] and metallosupramolecular [5] chemistry. In the former field, imidazoles mimic the side chain of histidine and are valuable in biological modeling. Metalloenzyme synthetic models target the enzyme active site structure, spectroscopy, and mechanism of action. Further, bioinorganic models may also lead to compounds which mimic enzyme function and provide new reagents or catalysts for practical application. In the latter field, the presence of both donor atoms to metal ions and hydrogen bond donors within imidazoles, combined with the *π*-excessive character of the 5-membered heterocyclic ring, can lead to intermolecular assembly of metal complexes through ligand-ligand or ligand-inorganic anion interactions. In spite of the enormous scientific literature on metal complexes with simple imidazoles as ligands, there is in fact relatively little known about the coordination and metallosupramolecular chemistry of heavily substituted imidazoles [1]. 

**Scheme 1:**
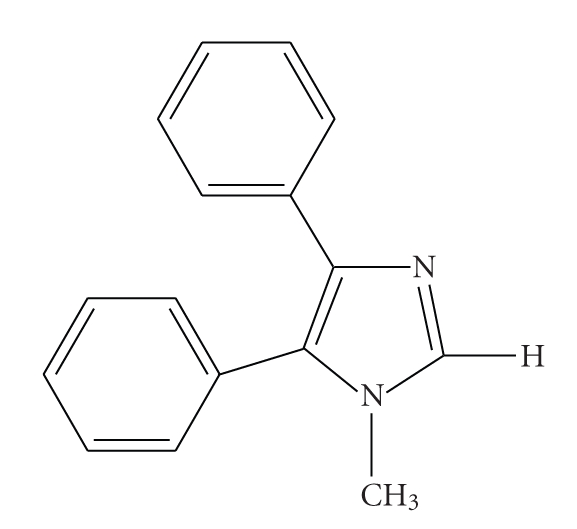
Drawing of the ligand 1-methyl-4,5-diphenylimidazole.

It is well established nowadays that the most prominent intermolecular interactions responsible for the supramolecular organization of metal complexes are hydrogen bonds and *π*-*π* stacking interactions [6–11]. With this in mind, an investigation has recently been initiated to determine the crystal structures of a designed series of transition metal complexes using heavily substituted imidazole ligands aiming at understanding the relative strength and the way in which these interactions control the noncovalent assembly of molecular building blocks in supramolecular systems [12]. In particular, 1-methyl-4,5-diphenylimidazole (L) (Scheme 1), a monodentate ligand capable of forming *π*-*π* interactions, has been selected to initiate our studies. So far, there have been only few studies on the coordination chemistry of L [13, 14]. The general reaction system currently in use involves M^II^/X^−^/L [M^II^ = Co, Ni, Cu, Zn; X^−^ = Cl^−^, Br^−^, I^−^, RCO_2_^−^, NO_3_^−^, NCS^−^, N_3_^−^] in various solvents and the first Co(II) and Zn(II) complexes have already been reported [12]. 

In this study we present our results on the M^II^/PhCO_2_^−^/L [M^II^ = Co, Ni, Cu, Zn] system. So far, two new complexes, namely, [Zn(O_2_CPh)_2_(L)_2_]·2MeOH (**1**·2MeOH) and [Ni_2_(O_2_CPh))_4_(L)_2_]·2MeCN (**2**·2MeCN) have been synthesized and characterized by elemental analyses, IR spectra, and single-crystal X-ray analysis.

## 2. Experimental

### 2.1. Materials and Instruments

Chemicals (reagent grade) were purchased from Merck and Alfa Aesar. All manipulations were performed under aerobic conditions using materials and solvents as received; water was distilled in-house. The ligand 1-methyl-4,5-diphenylimidazole (L) was synthesized as already described in a previous work [15]. Microanalyses (C, H, N) were performed by the University of Ioannina (Greece) Microanalytical Laboratory using an EA 1108 Carlo Erba analyzer. IR spectra were recorded on a Perkin-Elmer PC 16 FT-IR spectrometer with samples prepared as KBr pellets.

### 2.2. Compound Preparation

#### 2.2.1. Preparation of [Zn(O_2_CPh)_2_(L)_2_]·2MeOH (**1**·2MeOH)

This compound was synthesized by a solvothermal reaction of L (0.18 g, 0.75 mmol) and Zn(O_2_CPh)_2_·2H_2_O (0.10 g, 0.30 mmol) in MeOH (8 mL). The reaction mixture was loaded into a Teflon-lined stainless steel autoclave with inner volume of 20 mL, and then the sealed autoclave was heated under autogenous pressure at 150°C for 3 days. Upon slow (5°C/h) cooling to ambient temperature, colourless prismatic crystals of **1**·2MeOH (suitable for X-ray crystallography) appeared, which were collected by filtration, washed with cold EtOH (2 × 2 mL) and Et_2_O (2 × 5 mL), and dried in air; yield ca. 40% (based on the metal). A sample for crystallography was maintained in contact with the mother liquor to prevent the loss of lattice solvent* Anal. Calc*. for **1**·2MeOH: C, 68.61; H, 5.53; N, 6.67%. C, 68.30; H, 5.41; N, 6.88%. IR data (KBr, cm^−1^): 3446 (mb), 3130 (m), 3054 (m), 2924 (w), 1624 (s), 1570 (s), 1520 (s), 1484 (m), 1446 (m), 1366 (s), 1256 (m), 1196 (m), 1174 (w), 1126 (m), 1072 (m), 1024 (m), 1000 (w), 978 (m), 920 (m), 838 (m), 788 (s), 774 (s), 744 (sh), 720 (s), 700 (s), 680 (m), 650 (m), 580 (m), 512 (w).

#### 2.2.2. Preparation of [Ni_2_(O_2_CPh))_4_(L)_2_]·2MeCN (**2**·2MeCN)

A pale yellow solution of L (0.29 g, 1.25 mmol) in MeCN/CH_2_Cl_2 _(30 mL, 1 : 1 v/v) was treated with solid Ni(O_2_CPh)_2_·2H_2_O (0.17 g, 0.50 mmol). The resulting green slurry was stirred at ambient temperature for 20 min. The solution was filtered and the green filtrate was left undisturbed in a closed vial at room temperature. After 15 days, light-green crystals of **1**·2MeCN suitable for X-ray analysis formed were collected by filtration, washed with cold EtOH (2 × 2 mL) and Et_2_O (2 × 5 mL), and dried in air. Yield ca. 60% (based on the metal). A sample for crystallography was maintained in contact with the mother liquor to prevent the loss of lattice solvent. *Anal. Calc*. for **2**·2MeCN: C, 66.69; H, 4.73; N, 7.29%. Found: C, 66.81; H, 4.40; N, 7.38%. IR data (KBr, cm^−1^): 3134 (m), 3060 (m), 1626 (s), 1570 (s), 1522 (s), 1492 (w), 1444 (sh), 1418 (s), 1402 (s), 1254 (w), 1202 (m), 1174 (w), 1072 (m), 1024 (m), 978 (m), 922 (w), 842 (w), 786 (m), 776 (sh), 720 (s), 700 (s), 682 (m), 648 (m), 538 (w), 476 (m).

### 2.3. X-Ray Crystallography

Selected single crystals of **1**·2MeOH and **2**·2MeCN were covered with Paraton N oil and mounted on the tip of a glass capillary. X-ray data for both compounds were collected (*ω*-scans) on an Oxford Diffraction Xcalibur diffractometer under a flow of nitrogen gas at 100(2) K (Mo*K* *α* radiation). For data collection and reduction the CrysAlis CCD and RED packages were employed [16], respectively. The reflection intensities were corrected for absorption (multiscan method), the structures were solved by direct methods with SIR92 [17] and refined by full-matrix least-squares on *F* ^2^ with SHELXL-97 [18]. All nonhydrogen atoms were refined anisotropically. All hydrogen atoms bound to carbon atoms were introduced at calculated positions applying the riding model [C(*s* *p* ^2^)–H and C(*s* *p* ^3^)–H 0.93 and 0.96 Å, respectively; *U*_iso_(H) = 1.2*U*_eq_(C) (1.5 for C*s* *p* ^3^ methyl groups) of their parent C atom]. The hydroxyl hydrogen atoms of the solvent molecules in **1**·2MeOH were located by difference maps and their positions were refined isotropically [*U*_iso_(H) = 1.5*U*_eq_(O)] applying a soft distance restraint. All geometric calculations were carried out using WINGX [19], PLATON [20], and MERCURY [21] packages; molecular graphics were prepared with DIAMOND [22]. Details of the data collection and refinement are summarized in Table 1.

CCDC-771769 and CCDC-771770 contain the crystallographic data for **1**·2MeOH and **2**·2MeCN, respectively. These data can be obtained free of charge via http://www.ccdc.cam.ac.uk/conts/retrieving.html, or from the Cambridge Crystallographic Data Centre, 12 Union Road, Cambridge CB2 1EZ, UK; fax: (+44) 1223-336-033; or e-mail: deposit@ccdc.cam.ac.uk.

## 3. Results and Discussion

### 3.1. Synthetic Comments and IR Spectra

The reactions that led to complexes **1** and **2** can be represented by the stoichiometric equation (1)


(1)
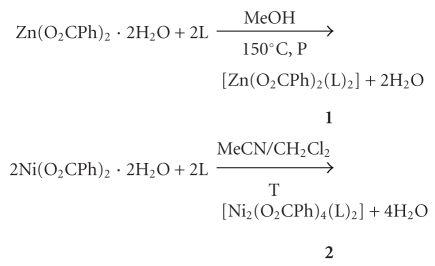



Two features of the reactions represented by (1) deserve brief comments. First, complex **1** could be crystallized [in the form of the bis(methanol) solvate] only under solvothermal conditions. Solvothermal techniques [23] allow the application of high temperatures to reactions in relatively low boiling solvents and are an excellent method for the preparation of pure, crystalline products. Second, despite the excess of the ligand (L : Ni^II^ = 2.5), only the dinuclear 1 : 1 complex **2** could be prepared. The steric bulk of both PhCO_2_^−^ and L seems to disfavor the isolation of a six-coordinate [Ni(O_2_CPh)_2_(L)_2_] molecule with chelating benzoato ligands. The preparation of the 5-coordinate dinuclear complex **2** can be partly attributed to the small tendency of Ni^II^ to form tetrahedral species. 

The IR bands of L [13] do not shift significantly in the spectra of **1** and **2**. The *ν*_as_(CO_2_) band is difficult to assign in the spectra due to the appearance of various stretching vibration and the *δ*_as_(CH_3_) mode in the 1630–1420 cm^−1^ region; thus, the application of the spectroscopic criterion of Deacon and Phillips [24] seems impossible [25]. The bands at 1366 and 1402 cm^−1^ in the spectra of **1** and **2**, respectively, can safely be assigned to the symmetric carboxylate stretching mode, *ν*_s_(CO_2_), of the benzoato ligands [24].

### 3.2. Description of the Structures

**Figure 1:**
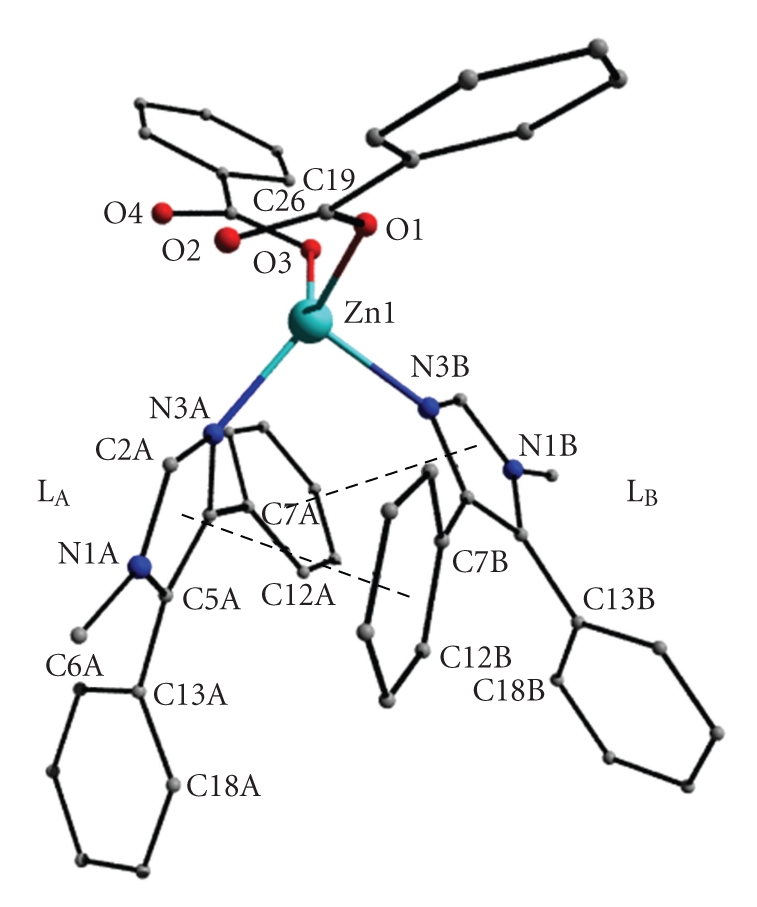
A partially labeled plot of complex **1**·2MeOH. The methanol molecules and the hydrogen atoms have been omitted for clarity. The intramolecular *π*-*π* interactions between the two ligands L_A_ and L_B_ are shown with dashed lines. Ring numeration: A1: N1A–C2A–N3A–C4A–C5A; A2: C7A to C12A; A3: C13A to C18A; B1: N1B–C2B–N3B–C4B–C5B; B2: C7B to C12B, B3: C13B to C18B.

**Figure 2:**
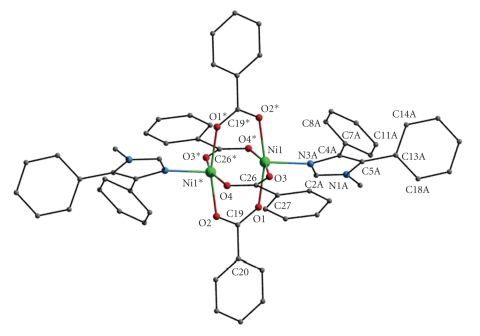
A partially labeled plot of the dinuclear complex **2**·2MeCN. The acetonitrile molecules and the hydrogen atoms have been omitted for clarity. Asterisks are used for symmetry related (1/2 − *x*, 1/2 − *y*, 1 − *z*) atoms.

The molecular structures of complexes **1**·2MeOH and **2**·2MeCN are shown in Figures 1 and 2, respectively, selected bond lengths, angles, and torsion angles are listed in Table 2. To facilitate comparison, both compounds have the same (where applicable) atom, ring, and ligand numbering (ligand L_A_: rings A1, A2 and A3; ligand L_B_: rings B1, B2 and B3; see Figure 1). 

Complex **1**·2MeOH consists of neutral mononuclear [Zn(O_2_CPh)_2_(L)_2_] molecules and methanol molecules in the lattice in a 1 : 2 ratio; it crystallizes in the monoclinic space group *P*2_1_/*n*. The two benzoate ions coordinate to Zn(II) in a monodentate fashion; this results in a distorted tetrahedral environment about the Zn(II) centre comprising two benzoate oxygen atoms and two pyridine-type, imidazole nitrogen donor atoms from the two 1-methyl-4,5-diphenylimidazole (L) molecules. Both benzoate species are planar. The L_A_ and L_B_ ligands of the complex are “antiparallel” with their methyl groups pointing at opposite directions. The overall conformation of L_A_ and L_B_ is similar. The angle between the mean planes of the phenyl rings A2/A3 and B2/B3 is 70.5(1)° and 67.0(1)°, respectively. Moreover, the imidazole ring A1 is facing the phenyl ring B2 [10.5(1)°] and, similarly, the imidazole ring B1 is facing the phenyl ring A2 [4.9(1)°] forming weak intramolecular interligand *π*-*π* interactions among those pairs of rings (Table 3). It seems that steric effects and the distorted tetrahedral geometry of the Zn(II) centre [N3A–Zn1–N3B = 96.6(1)°] facilitate those *π*-*π* interactions. Similar intramolecular *π*-*π* interactions between L_A_ and L_B_ have also been reported for Pd^II^ [14], and Co^II^ and Ni^II^ [12] complexes with L, in a series of analogous complexes of other divalent metals with L [26] as well as in Cu^II^ and Zn^II^ complexes of 2-[2′-(4′,6′-di-*tert*-butylhydroxyphenyl)]-4,5-diphenylimidazole [27, 28]. This structural feature records a preferable mode of packing between adjacent ligands bearing the 4,5-diphenylimidazole moiety and provides stabilization within the complex; it also supports the suitability of the ligand L as a crystal engineering tool, namely, its effect, through the *π*-*π* interactions, in the assembly and packing of complexes in inorganic supramolecular chemistry [29, 30]. The length of the C–C and C–N bonds of the imidazole groups, for both **1**·2MeOH and **2**·2MeCN, are as expected in [31]; the Zn–N and Zn–O bond lengths are normal for this kind of compound. 

**Figure 3:**
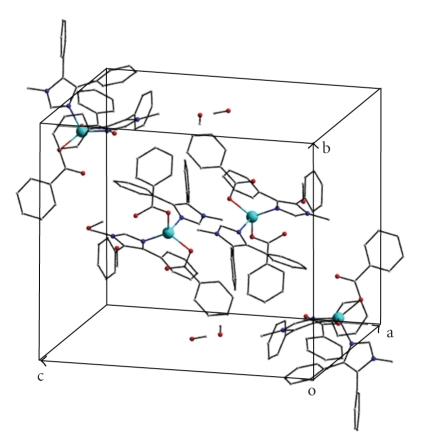
View of the crystal packing of complex **1**·2MeOH. The hydrogen atoms have been omitted for clarity. Zn: turquoise; O: red; N: blue; C: grey.

One of the methanol molecules (O5) in the lattice is involved in two strong intramolecular O–H⋯O hydrogen bonds to the noncoordinated oxygen atom (O2) of one benzoate and to the second methanol (O6) molecule. There is also a weak C–H⋯O(benzoate) interaction involving the noncoordinated oxygen atom (O4) of the other benzoato ligand. The packing of the molecules in the crystal lattice proceeds through normal van der Waals contacts and some weak intermolecular C–H⋯O interactions contributing to the supramolecular assembly of the structure (Table 4). It is known that C–H⋯O bonds could play a role in the organization of crystal packing, especially when classic hydrogen bonding is absent [32–35]. A view of the crystal packing of complex **1**·2MeOH is shown in Figure 3. 

The dinuclear paddle-wheel type complex **2**·2MeCN, [Ni_2_(O_2_CPh))_4_(L)_2_]·2MeCN, crystallizes in the monoclinic space group *C*2/*c* with two solvate acetonitrile molecules. The asymmetric unit comprises half molecule of the complex and one acetonitrile molecule, and the structure is generated by inversion at the midpoint of the Ni⋯Ni distance. The four bidentate benzoate groups bridge the two Ni ions in a paddle-wheel arrangement about the Ni⋯Ni axis. Thus, each Ni^II^ atom is penta-coordinated exhibiting a square pyramidal geometry with the apex occupied by the pyridine-type, imidazole nitrogen donor atom (N3A) of one monodentate 1-methyl-4,5-diphenylimidazole ligand. The Ni to apical N3A atom distance is 2.017(2), the four Ni–O(benzoate) bond lengths range from 2.008(2) to 2.039(2) Å, and the Ni⋯Ni–N3A angle is 160.8(1)°. The Ni atom lies 0.266(1) Å out of the least-squares basal plane towards N3A atom. The Ni⋯Ni distance is 2.734(1) Å, shorter than the maximum distance of ~3.5 Å that the tetracarboxylate paddle-wheel motif can accommodate for metal-metal separations. The acetonitrile molecule is linked to the complex via a weak C–H⋯O1(benzoate) interaction (Table 4). The bridged dinuclear structure M_2_(*η* ^1^ : *η* ^1^ : *μ*-O_2_CR)_4_(ligand)_2_, first documented in 1953 for copper(II) acetate monohydrate, is ubiquitous in modern coordination chemistry [36]. It is found not only for carboxylates of many transition elements, but also for dimers containing a wide variety of other triatomic bridging ligands. This structural type is associated with a spectrum of metal-metal interactions ranging from no interactions, weak or moderate spin-pairing in the copper(II) carboxylates, various orders of metal-metal bonding, to the “super-short” metal-metal bonds (M–M < 2 Å). The axial groups are normally monodentate ligands but they may represent interdimer association into a polymeric structure or may be absent. 

It should be mentioned that attempts were made to prepare metal(II)-benzoate complexes with the 1-methyl-4,5-diphenylimidazole ligand for the divalent metals Co, Ni, Cu, and Zn, varying the factors that could affect the self-assembly of supramolecular architectures (such as the solvents used, temperature, counter-ion, the ligand-to-metal ratio, method of preparation, etc.). However, our trials yielded only the present two crystalline materials. It seems that the capability of the benzoate group to adopt different ligation modes plays, at least in the present case, a role in the formation of different coordination structural types.

## 4. Conclusions

The use of 1-methyl-4,5-diphenylimidazole ligand (L) in reactions with Zn(O_2_CPh)_2_·2H_2_O and Ni(O_2_CPh)_2_·2H_2_O has yielded the mononuclear [Zn(O_2_CPh)_2_(L)_2_]·2MeOH complex (**1**·2MeOH) and the dinuclear [Ni_2_(O_2_CPh))_4_(L)_2_]·2MeCN (**2**·2MeCN) compound. The different benzoate binding mode to the metals used, monodentate in the former and bidentate in the latter complex, has led to two different coordination geometries for the two divalent metals. The characteristic structural pattern [12] of the intramolecular *π*-*π* interactions between aromatic rings of adjacent 4,5-diphenylimidazole moieties of the two L ligands is also present in the structure of **1**·2MeOH and contributes to the stability and rigidity of the structure.

## Figures and Tables

**Table 1:** Crystal data and refinement parameters for complexes **1**·2MeOH and **2**·2MeCN.

	**1**·2MeOH	**2**·2MeCN
Empirical formula	C_48_H_46_N_4_O_6_Zn	C_64_H_54_N_6_O_8_Ni_2_
Formula weight (g mol^−1^)	840.26	1152.55
Temperature	100(2)	100(2)
Wavelength	0.71073	0.71073
Crystal system	monoclinic	Monoclinic
Space group	*P*2_1_/*n*	*C*2/*c*
*a* (Å)	13.8301(2)	29.7994(16)
*b* (Å)	16.2359(2)	10.4438(6)
*c *(Å)	18.6267(3)	18.1018(11)
*β* (°)	94.075(2)	98.745(6)
*V* (Å^3^)	4171.94(10)	5568.1(6)
*Z*	4	4
Density (calculated) (g cm^−3^)	1.338	1.375
Absorption coefficient (mm^−1^)	0.644	0.739
*F*(000)	1760	2400
Crystal size (mm)	0.38 × 0.26 × 0.18	0.22 × 0.21 × 0.08
Colour, habit	colorless, prism	light green, plate
*θ* range for data collection (°)	3.07 to 30.29	3.15 to 30.39
Index ranges	−18 ≤ *h* ≤ 12	−36 ≤ *h* ≤ 21
	−22 ≤ *k* ≤ 22	−12 ≤ *k* ≤ 12
	−25 ≤ *l* ≤ 25	−20 ≤ *l* ≤ 21
Reflections collected/unique (*R*_int _)	36095/11013 (0.0304)	18454/5154 (0.0790)
Observed reflections [*I* > 2*σ*(*I*)]	7850	2906
Data/restraints/parameters	11013/2/542	5154/0/363
Goodness-of-fit on *F * ^2^	0.962	0.805
Final *R*_1_^a^, *w* *R*_2_^b^ [*I* > 2*σ*(*I*)]	0.0324, 0.0788	0.0392, 0.0591
Mean and max shift/error	0.000 and 0.002	0.000 and 0.001
Largest diff. peak and hole (e Å^−3^)	0.503 and −0.457	−0.259/0.666

^a^ *R*_1_ = Σ | |*F*_*o*_| − |*F*_*c*_| | /Σ|*F*_*o*_|.

^b^ *w* *R*_2_ = {Σ*w*(*F*_o_^2^−*F*_c_^2^)^2^/Σ*w*(*F*_o_^2^)^2^}^1/2^.

**Table 2:** Selected interatomic distances (Å), angles and torsion angles (°) for **1**·2MeOH and **2**·2MeCN.

Compound	**1**·2MeOH	**2**·2MeCN
M	Zn	Ni
M⋯M^i^		2.734(1)
M–N3A	2.007(1)	2.017(2)
M–N3B	2.065(1)	
M–O1	1.947(1)	2.015(2)
M–O3	1.950(1)	2.039(2)
M–O2		2.008(2)
M–O4		2.026(2)

N3A–M–N3B	96.6(1)	
N3A–M–O1	123.4(1)	91.9(1)
N3A–M–O2		103.3(1)
N3A–M–O3	123.9(1)	98.2(1)
N3A–M–O4		96.7(1)
N3B–M–O1	103.0(1)	
N3B–M–O3	97.2(1)	
O1–M–O3	105.7(4)	88.0(1)
O1–M–O4		89.9(1)
O2–M–O3		90.7(1)
O2–M–O4		87.4(1)
C19–M–C26	112.46(4)	

A2–A3*	70.5(1)	61.9(1)
B2–B3*	67.0(1)	
C4A–C5A–C13A–C14A	−57.8(2)	−53.0(4)
C4B–C5B–C13B–C14B	−56.8(2)	
C5A–C4A–C7A–C12A	−46.6(2)	−40.5(4)
C5B–C4B–C7B–C12B	−37.3(2)	

*Angle between the mean-planes of the named phenyl rings (see Figure 1).

Symmetry codes: (i) 1/2 − *x*, 1/2 − *y*, 1 − *z*.

**Table 3:** Geometrical details (Å, °) of the intramolecular *π*-*π* interactions between L_A_ and L_B_ ligands for complex **1**·2MeOH.

	Rings	Distance/Angle
Distance between ring centroids	A1–B2	3.547(1)
	B1–A2	3.622(1)
Perpendicular distance between ring planes	A1–B2	3.437(1)
	B1–A2	3.363(1)
Centroid offset	A1–B2	0.876(1)
	B1–A2	1.346(1)
Dihedral angle between ring mean-planes	A1–B2	10.5(1)
	B1–A2	4.9(1)

**Table 4:** Hydrogen-bond geometries for **1**·2MeOH and **2**·2MeCN (Å, °).

D–H⋯A	D–H	H⋯A	D⋯A	D–H⋯A
*Complex * **1·**2*MeOH *				
O6–H6⋯O5	0.855(19)	1.916(19)	2.764(2)	172(2)
O5–H5⋯O2^i^	0.847(17)	1.913(17)	2.738(2)	165(2)
C8B–H8B⋯O1	0.93	2.50	3.282(2)	142
C6B–H6B3⋯O1^ii^	0.96	2.432	3.370(2)	166
C33–H33B⋯O4^i^	0.96	2.52	3.423(2)	157

*Complex * **2**·2*MeCN *				
C8A–H8A⋯O2	0.93	2.30	3.151(3)	152
C34–H34C⋯O1^iii^	0.96	2.50	3.371(4)	151

Symmetry codes: (i) −1 + *x*, −1 + *y*, *z*; (ii) 2 − *x*, 2 − *y*, −*z*; (iii) *x*, 1 + *y*, *z*.
